# Use of high-content imaging to quantify transduction of AAV-PHP viruses in the brain following systemic delivery

**DOI:** 10.1093/braincomms/fcab105

**Published:** 2021-05-17

**Authors:** Edward J Smith, Pamela P Farshim, Rachel Flomen, Samuel T Jones, Sean J McAteer, Benjamin E Deverman, Viviana Gradinaru, Gillian P Bates

**Affiliations:** 1 Huntington’s Disease Centre, Department of Neurodegenerative Disease, UK Dementia Research Institute at UCL, Queen Square Institute of Neurology, University College London, London WC1N 3BG, UK; 2 The Stanley Center for Psychiatric Research at the Broad Institute of MIT and Harvard, Cambridge, MA 02142, USA; 3 Division of Biology and Biological Engineering, California Institute of Technology, Pasadena, CA 91101, USA

**Keywords:** gene therapy, AAV-PHP viruses, CNS transduction, high-content imaging, *Ly6a* inbred strain variants

## Abstract

The engineering of the AAV-PHP capsids was an important development for CNS research and the modulation of gene expression in the brain. They cross the blood brain barrier and transduce brain cells after intravenous systemic delivery, a property dependent on the genotype of *Ly6a*, the AAV-PHP capsid receptor. It is important to determine the transduction efficiency of a given viral preparation, as well as the comparative tropism for different brain cells; however, manual estimation of adeno-associated viral transduction efficiencies can be biased and time consuming. Therefore, we have used the Opera Phenix high-content screening system, equipped with the Harmony processing and analysis software, to reduce bias and develop an automated approach to determining transduction efficiency in the mouse brain. We used R Studio and ‘gatepoints’ to segment the data captured from coronal brain sections into brain regions of interest. C57BL/6J and CBA/Ca mice were injected with an AAV-PHP.B virus containing a green fluorescent protein reporter with a nuclear localization signal. Coronal sections at 600 μm intervals throughout the entire brain were stained with Hoechst dye, combined with immunofluorescence to NeuN and green fluorescent protein to identify all cell nuclei, neurons and transduced cells, respectively. Automated data analysis was applied to give an estimate of neuronal percentages and transduction efficiencies throughout the entire brain as well as for the cortex, striatum and hippocampus. The data from each coronal section from a given mouse were highly comparable. The percentage of neurons in the C57BL/6J and CBA/Ca brains was approximately 40% and this was higher in the cortex than striatum and hippocampus. The systemic injection of AAV-PHP.B resulted in similar transduction rates across the entire brain for C57BL/6J mice. Approximately 10–15% of all cells were transduced, with neuronal transduction efficiencies ranging from 5% to 15%, estimates that were similar across brain regions, and were in contrast to the much more localized transduction efficiencies achieved through intracerebral injection. We confirmed that the delivery of the AAV-PHP.B viruses to the brain from the vasculature resulted in widespread transduction. Our methodology allows the rapid comparison of transduction rates between brain regions producing comparable data to more time-consuming approaches. The methodology developed here can be applied to the automated quantification of any parameter of interest that can be captured as a fluorescent signal.

## Graphical Abstract

**Graphical Abstract:**
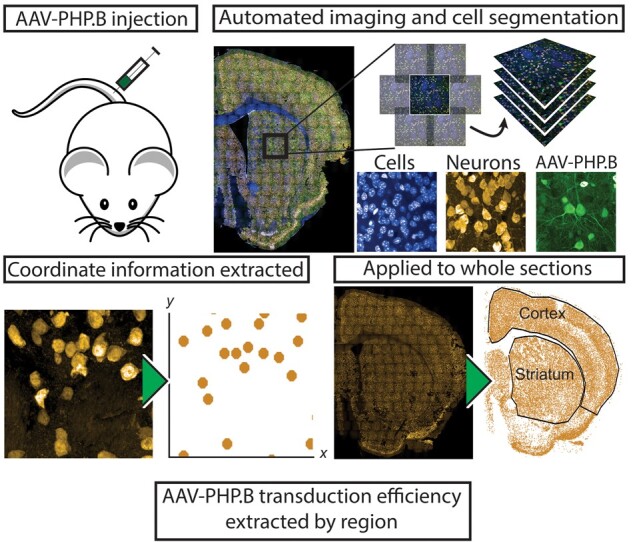

Abbreviated summary

Smith et al. used the Opera Phenix high-content screening system and R Studio to automate the quantification of viral transduction efficiency in the mouse brain. They show that the systemic delivery of AAV-PHP.B viruses results in widespread and even transduction throughout the brain and brain regions.

## Introduction

Adeno-associated viral (AAV) vectors are commonly used vehicles in gene therapy research for neurological disease. Considerable progress has been made in efforts to engineer novel recombinant AAV capsids, to improve tissue tropism and enhance CNS transduction efficiency in preclinical investigation settings. As a result, many natural and engineered isolates have been described.^1^^,^^2^ With the advent of more diverse gene therapy vectors, there is an urgent need to develop accurate and rapid quantification techniques that can be applied to measure viral transduction in complex tissues.

A Cre-recombination-based AAV targeted evolution (CREATE) platform was used to generate variants of AAV9: AAV-PHP.B,^3^ and the enhanced form AAV-PHP.eB ^4^ that penetrate the blood brain barrier (BBB) more effectively than AAV9 following systemic delivery. They have improved CNS tropism, resulting in widespread transduction of neurons and glia throughout the mouse brain. Although the transduction efficiency of these variants is up to 40-fold higher than that of AAV9, the transduction levels can be variable.^3–6^ Moreover, the transduction capability of AAV-PHP variants has been shown to be both strain- and species-specific.^7–11^ In mice, the CNS tropism of AAV-PHP.B and AAV-PHP.eB is absent in some inbred strains e.g. BALB/cJ, and the ability of these viruses to cross the BBB is dependent on single nucleotide variants in the *Ly6a* gene, which encodes the receptor for the AAV-PHP capsids.^7^^,^^8^^,^^11^ These limitations have been circumvented by the further development of the CREATE platform and the identification of capsid variants that can transduce the CNS broadly, with different biases towards neurons, glia or vascular cells and that cross the BBB for a diverse set of murine inbred strains.^5^

Whilst there have been significant advances in expanding the repertoire of AAV capsids with specific and diverse tropisms, the quantification of transduction efficiencies in complex tissues remains a challenge. Although current approaches enable the acquisition of high-resolution images of immunohistochemically labelled tissues, quantification methodologies remain biased and time-consuming. High-Content Screening (HCS) systems have been used in *in vitro* studies providing a robust, reliable platform for morphological and cellular quantification.^12–16^ Whilst its use *in vitro* has been well documented, there are only a handful of studies that have explored its application *in situ* or *ex vivo.* HCS platforms have been used to determine the percentage of microglia and astrocytes in the mouse brain in 1/200th of the time taken by manual analytical techniques.^17^ Carty and colleagues demonstrated that such techniques could be applied to the quantification of the number and location of inclusion bodies in the brains of a mouse model of Huntington’s disease.^18^ More recently, HCS has been used to quantify gene expression patterns across distinct astrocytic populations in different cortical regions at the single cell level.^19^

We have used the Opera Phenix HCS system coupled with Harmony analytical software to develop an automated analysis tree to quantify viral transduction in the mouse brain. We used the AAV-PHP.B virus to deliver a green fluorescent protein (GFP) reporter to the CNS of C57BL/6J mice by intravenous injection. Brain sections were stained with Hoechst to label all cell nuclei, and immunostained for NeuN to label neurons and with GFP to label transduced cells; allowing the percentage of neurons, transduced cells and transduced neurons to be quantified throughout the entire brain. We developed R-scripts by which the brain could be segmented into specific brain regions and quantified the neuronal percentages and transduction efficiencies for cortex, striatum and hippocampus. The transduction efficiencies were consistent between all coronal sections and brain regions for a given mouse indicating that the systemic delivery of AAV-PHP.B results in an even transduction efficiency throughout the brain. As an independent measure of biodistribution, we applied an absolute qPCR assay to measure viral genome copy number (VGCn) in blood and tissues two hours post-dosing with AAV-PHP.eB. We found that the virus enters the brain very rapidly and that levels in most brain regions were higher than those in liver.

## Materials and methods

### Mouse breeding and maintenance

All animal procedures were performed in accordance with the Animals (Scientific Procedures) Act 1986 and were approved by the University College London Ethical Review Process Committee. C57BL/6J (C57BL/6JOlaHsd) and CBA/Ca (CBA/CaOlaHsd) mice were from Envigo, Netherlands. Mouse husbandry was as previously described.^20^ Mice were housed in individually ventilated cages with up to five mice per cage, dependent on sex, with Aspen Chips 4 Premium bedding (Datesand) and environmental enrichment, which included chew sticks and play tunnel (Datesand). On arrival from the supplier, mice were handled daily and acclimatised to the facility for one week prior to the initiation of experiments. Mice had unrestricted access to food (Teklad global 18% protein diet, Envigo) and water. The temperature was regulated at 21°C ± 1°C and animals were kept on a 12 h light/dark cycle. The animal facility was barrier-maintained and quarterly non-sacrificial Federation of European Laboratory Animal Science Associations (FELASA) screens found no evidence of pathogens.

### Genotyping

Tail-tip DNA was prepared using high salt extraction as previously described.^21^ For the *Ly6a* variant, 25 ng DNA was amplified in two separate reactions using either C57insF (5′-CTGGAATTAGGAATGGTTGTGTG-3′) and C57insR (5′-TTGTTCTTTACTTTCCTTGTTTGAGAAT-3′) or CBAinsF2 (5′-TAGGAATGGTTGTAAACCAGG-3′) and CBAinsR2 (5′-CCTCCATTGGGAACTGCTG-3′) primers with Dream-Taq master mix (Thermo Fisher Scientific). Cycling conditions were 3 min at 95°C, 35 × (30 s at 95°C, 30 s at 60°C, 60 s at 72°C), 5 min at 72°C.

### AAV viruses

The ssAAV-PHP.B-CAG-NLS-GFP reporter was prepared as previously described.^3^ The ssAAV-PHP.eB-CAG-NLS-GFP reporter was a kind gift from Miguel Estevez (University of Massachusetts). The viral stocks had titres of 2.1 × 10^13^ VG/ml and 2.29 × 10^12^ VG/ml and were stored aliquoted at −80°C.

### Viral injections

For the HCS, ssAAV-PHP.B-CAG-NLS-GFP was administered to three C57BL/6J and three CBA/Ca mice at 4 weeks of age (two male and one female in each case). Mice were placed in a heated recovery chamber (∼40°C ± 2) for 10 min prior to each injection to dilate veins and administered with 1 × 10^11^ vg/mouse [in 200 μl sterile phosphate buffered saline (PBS)] via the lateral caudal vein. At 1 h post-injection, the animal was placed back in the heated chamber before collecting up to 200 μl of blood into a lithium-heparin coated tube, which was placed on ice.

For the tissue biodistribution study, ssAAV-PHP.eB-CAG-NLS-GFP was administered to eight C57BL/6J mice at 6–7 weeks of age (four male and four female) and PBS to two C57BL/6J mice (one male and one female) as described above. At 1 h post-injection, the animal was placed back in the heated chamber before collecting up to 200 μl of blood into a lithium-heparin coated tube on ice. After 2 h, mice were euthanized, blood was collected by cardiac puncture and mice were perfused with cold heparinized PBS. Brain regions and liver were dissected and immediately snap frozen in liquid nitrogen before storage at −80°C.

### Quantitation of viral genome copy number in blood and tissues

Recombinant AAV DNA was isolated from blood using the HiPure Viral Nucleic Acid Kit (Roche) and from tissues by high salt extraction.^21^ The plasmid pAAV-pCAG-EGFP (Addgene #37825) was used for standard curve generation. DNA concentrations were determined using a Qubit v2 fluorimeter with Qubit dsDNA HS and BR assay kits (Thermo Fisher Scientific). An absolute quantitative assay was developed based on the previously described free-inverted terminal repeat (ITR) method^22^ using a TaqMan real-time quantitative PCR (qPCR) assay targeting the ITR sequence^23^ (Thermo Fisher Scientific). The pAAV-pCAG-EGFP plasmid DNA was linearized by *PvuII* digestion adjacent to both ITR sequences, purified using the QIAquick PCR purification kit (Qiagen) and a 10-fold serial dilution was prepared (1 × 10^8^ to 1 × 10^4^). A 10 μl reaction contained: 10 ng DNA, 900 nM primers, 250 nM probe, TaqMan Fast Advanced Master Mix (Life Technologies). Cycling conditions were 2 min at 50°C, 10 min at 95°C, 40 × (15 s at 95°C, 60 s at 60°C). Reactions were performed in triplicate using a CFX96 real-time PCR cycler (Bio-Rad) and the number of viral genomes in blood determined from the plasmid standard curve.

### Tissue preparation and immunohistochemistry

Three weeks post-injection, mice were euthanized by transcardial perfusion with cold heparinized PBS followed by cold 4% paraformaldehyde solution (Pioneer Research Chemical Ltd). Whole brains were harvested, post-fixed in 4% paraformaldehyde solution for 24 h, cryoprotected with gradient steps of 20% and 30% sucrose in 0.01 M PBS, embedded in O.C.T (CellPath Ltd) and stored at −80°C. Coronal brain sections were cut on a cryostat at 25 µm, and every other section, placed in 12 well plates, and stored free-floating in tissue protective solution (30% ethylene glycol, 25% glycerol and 0.05% sodium azide in PBS) at −20°C until required for staining.

Free-floating sections were washed three times in PBS for 15 min, permeabilised at room temperature for 10 min in 0.3% Triton in PBS and then blocked with 10% normal goat serum (in 0.3% Triton PBS) for 1 h at room temperature. The sections were incubated with the rabbit anti-GFP (1:1000; Life Technologies, A-11122) or mouse anti-NeuN (1:500, Invitrogen, MA5-33103) antibodies in fresh blocking solution overnight at 4°C. The next day, the sections were washed three times in PBS and incubated with either AlexaFluor488 goat anti-rabbit for GFP (1:500; Life Technologies, A-11034) or AlexaFluor594 goat anti-mouse for NeuN (1:500, Life Technologies, A-11032) in PBS for 2 h protected from light. Sections were washed three times in PBS and incubated with Hoechst 33342 in PBS (1:5000; Invitrogen, H3570) for 15 min in the dark, washed three times in PBS and mounted onto microscope slides. After air-drying, the sections were cover slipped using Vectashield anti-fade mounting solution (Vector Laboratories: H-1000).

Coronal brain sections were examined for GFP expression using a Nikon Eclipse A1R point scanning confocal microscope (10×/0.3 air objective) by combining an 8 × 8 field panel with a stitching procedure with 15% overlap. Images were acquired with 405 nm and 488 nm lasers using Nikon-Filter cubes with excitation filters of 450/50 or 525/50 and captured using a Nikon A1 camera in NIS-Elements AR Software. Representative images were then processed using Fiji (ImageJ, Image Analysis).

### *In vivo* high-content image acquisition, processing and analysis

For high-content imaging, every 12th section throughout the entire brain was immunostained. The staining procedure, as described above, was followed until the last three wash steps, at which point, each section was carefully mounted onto the surface of a 24-well glass bottom SensoPlate (Greiner Bio-One). Owing to scanning magnification restriction, only the central eight wells could be used. Residual PBS was aspirated and 200 μl of 0.5% low temperature gelling agarose (Sigma; A0701) at 30°C was added in a drop-wise fashion until the section was completely covered without air pockets. The plates were protected from light and stored at 4°C.

Automated image capture was performed using the Opera Phenix High-Content Screening (HCS) System (PerkinElmer Inc.) equipped with Harmony high-content processing and analysis software (version 4.82). Initial low resolution, low magnification (10×/0.3 Air objective, HH14000403) scout images were taken across the whole of each well to locate the sections within, using the Hoechst signal as the nucleus indicator. Higher magnification (40×/1.1 Water objective, HH14000422) images were then captured on a 16 bit sCMOS camers using 405 nm/488 nm/561 nm lasers with Phenix emission filters 435–515 nm, 500–550 nm, 570–630 nm to detect cell bodies (Hoechst, blue), neurons (NeuN, red/magenta) and transduced cells (GFP, green), respectively. Images were captured with a 10% overlap between adjacent images and in a series of 19 optical slices 1.2 μm apart through the *Z* axis.

To automate processing of the captured data, an analysis sequence was prepared using Harmony’s inbuilt analysis building blocks. Input images were not subjected to flatfield correction but did have brightfield correction applied. The stack processing features were applied to maximum projections, to incorporate data from images captured through the *Z*-axis. Individual cells within each captured slice were identified from the Hoechst signal after the Gaussian 3 px smoothing filter was applied. The ‘find nuclei building block’ [algorithm method C, common threshold (CT) 0.35, area > 15 μm^2^, splitting coefficient (SC) 7, individual threshold (IT) 0.4, contrast < 0.06] was used to identify cells that met the set criteria. The ‘find cells building block’ was then used to identify cells based on either the NeuN signal (algorithm method C, CT 0.1, area > 30 μm^2^, SC 4, IT 0.4, contrast < 0.1) or the GFP signal (algorithm method C, CT 0.1, area > 14 μm^2^, SC 7, IT 0.4, contrast < 0.18) independently. The user inputs to the machine learning algorithms were repeated to decrease the stringency of the GFP+ gating (algorithm method C, CT 0.1, area > 14 μm^2^, SC 7, IT 0.4, contrast < 0.18).

Advanced STAR morphology analysis was used to analyse the properties of the cells identified, based on geometric features as well as signal intensity and distribution. Researcher input was used to determine whether a signal was true or false, which was then used by the programme, via machine learning, to identify common features of true signals and to apply these to the analysis. This led to the gating of the NeuN and GFP signals to filter out false positives. These outputs were then applied together to identify populations of cells that had been transduced (GFP+), neuronal cells (NeuN+) and transduced neurons (NeuN+/GFP+).

Quantitative data were exported from Harmony for all eight sections on a plate as single .csv text files and imported into Excel (Microsoft). The data were filtered based on the placement of the sample in the wells, and the percentage of cells that were either NeuN+, GFP+, or NeuN+/GFP+ were then extracted separately and graphed for each individual brain coronal section. For the segmentation of individual brain regions, the spatial information of each individual cell, in relation to its position within the well, was extracted as x and y coordinates and saved as individual .csv files. These data were imported into R studio and plotted in the graphical package X11. The libraries ‘devtools’ and ‘gatepoints’ (https://github.com/wjawaid/gatepoints, last accessed October 2018) were loaded into R from the online coding repository Github. Gatepoints was used to freehand draw regions of interest (ROIs) onto the plotted data to allowed manual segmentation of brain regions, as previously defined.^24^ The anterior striatal boundary was considered to be from the first appearance of the genu of the corpus callosum, and the posterior, to be first evidence of the hippocampal formation. The dorsal and lateral boundaries consisted of the corpus callosum with the medial boundary being the lateral ventricle/internal capsule.^25^ NeuN+ cells were used as the template to create ROIs as they allowed better delineation of regional boundaries over Hoechst. Datapoints within the ROI were extracted for each region and segmented. ROIs were saved and reapplied to extract identical positional data for Hoechst and GFP. Data for the cortex, striatum and hippocampus were exported for NeuN+, GFP+, and NeuN+/GFP+ cells and graphed for individual structures in each brain section.

### Statistical analysis

Data were screened for outliers using Grubb’s Test (GraphPad software), and no outliers were identified. Linear regression analysis was performed in Excel (Microsoft).

### Data availability

The authors confirm that all data supporting the findings of this study are available within the article. Raw data and R-scripts will be shared by the corresponding author on request.

## Results

### AAV-PHP.B administration to C57BL/6J and CBA/Ca inbred strains of mice

**Figure 1:**
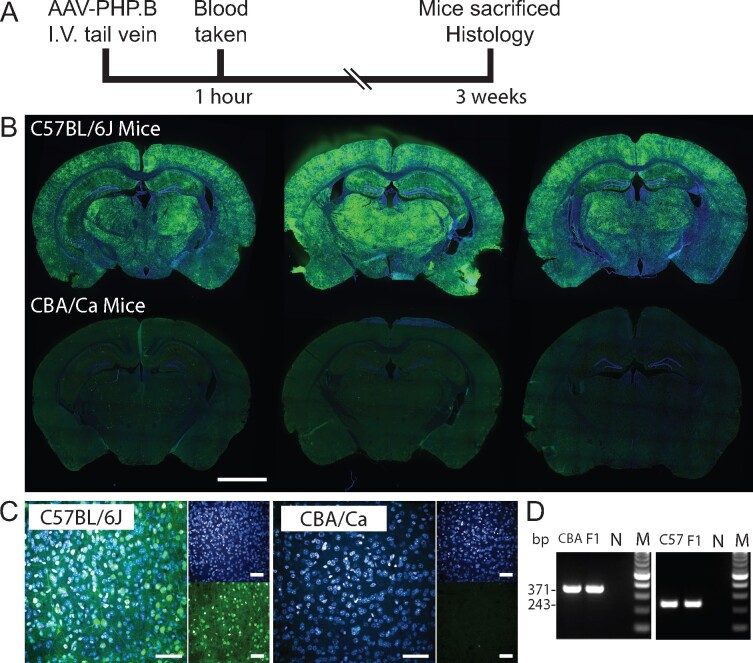
**Administration of the AAV-PHP.B virus to C57BL6/J and CBA/Ca mice**. (**A**) C57BL/6J and CBA/Ca mice were intravenously injected with ssAAV-PHP.B-CAG-NLS-GFP via the tail vein, blood was collected after 1 h and the mice were sacrificed 3 weeks post-injection. (**B**) Immunolabelling with an antibody to GFP produced an evenly distributed signal on the C57BL/6J brain sections, but not on those from CBA/Ca mice, scale bar = 2 mm. (**C**) The GFP signal was located in a subset of cell nuclei in the C57BL/6J brain sections, scale bar = 50 μm. (**D**) Genotyping for the *Ly6a* variant between CBA/Ca (CBA) and C57BL/6J (C57). The CBA/Ca assay provides a band of 371 bp and the C57BL/6J assay of 243 bp. F1 = mice heterozygous for the C57BL/6 and CBA variants. N = water, M = 100 bp ladder.

We used the ssAAV-PHP.B-CAG-NLS-GFP virus to develop a semi-automated method by which viral transduction in the brain could be quantified using high-content imaging. Three C57BL/6J and three CBA/Ca mice at four weeks of age were injected with 1 × 10^11^ ssAAV-PHP.B-CAG-NLS-GFP via the tail vein and blood was collected at 1 h post-injection. Mice were sacrificed after 3 weeks and their brains processed for immunohistochemistry (Fig. 1A). The brains were sectioned and immunostained with a GFP antibody to visualize transduced cells. Whilst an evenly distributed GFP signal could be detected in the C57BL/6J brains, confirming that the ssAAV-PHP.B-CAG-NLS-GFP virus had crossed the BBB, a GFP signal was absent from the brains of CBA/Ca mice (Fig. 1B). Visualization at the microscopic level confirmed that the GFP signal in the C57BL/6J brains was located in individual cells (Fig. 1C). Genotyping confirmed that the C57BL/6J mice were positive for the *Ly6a* variants that have been shown to be permissive for AAV-PHP viruses to cross the BBB whereas the CBA/Ca mice were not (Fig. 1D).^7^^,^^8^ Given that the virus had not entered the brains of the CBA/Ca mice, we used these as a negative control for viral transduction to determine background signal levels. Had we intended to quantify viral transduction of peripheral tissues, we would have needed to include PBS treated mice.

Before proceeding further, the precision of viral administration was verified using absolute quantification of viral genome copies (VGCn) in blood extracted from the tail vein at 1 h post-injection. Whilst the VGCn for the C57BL/6J mice was highly comparable [1.02 × 10^6^ ± 0.14 × 10^6^ (SD) VGCn/10 ng DNA], it was not possible to quantify viral genomes in blood from the CBA/Ca mice, as the blood clotted, despite being immediately placed in lithium heparin tubes, and DNA yields were low and of poor quality.

### Image capture and analysis

**Figure 2:**
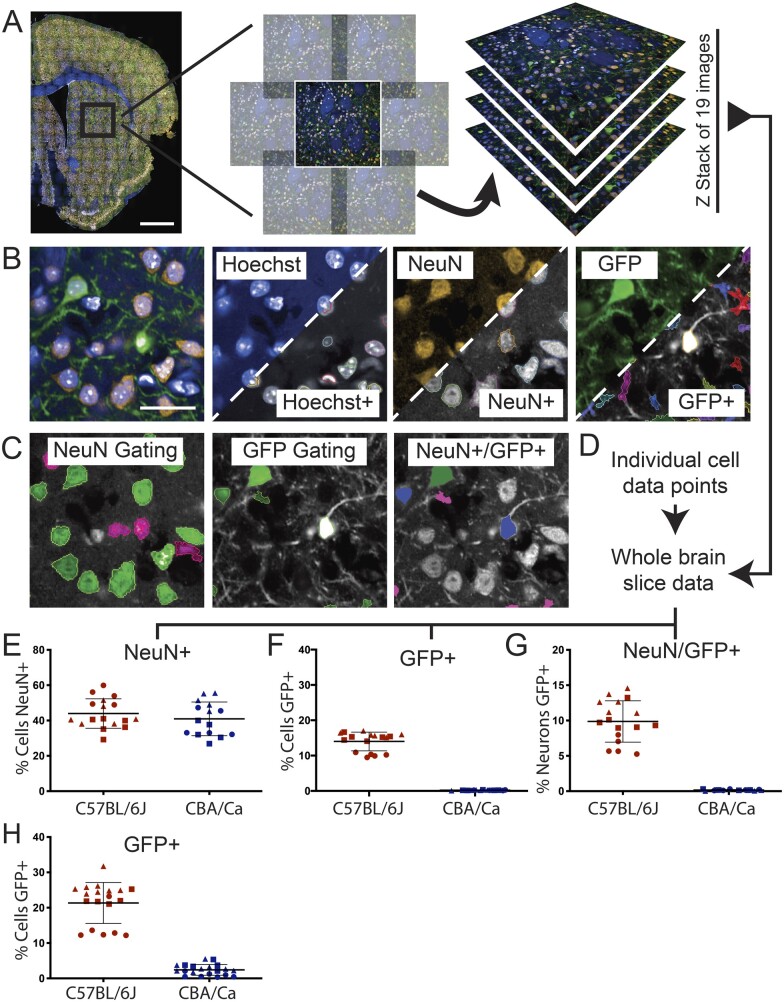
**Building an automated analysis tree to quantify AAV transduction**. (**A**) Brain sections were stained with Hoechst and immunostained for NeuN and GFP and images were captured on the Opera Phenix. Whole brain composites were generated by stitching together a panel of images with a 10% overlap. This was performed for 19 optical slices captured throughout the Z-plane at 1.2 μm intervals. (**B**) Each channel was analysed separately to identify positive signals for cell nuclei (Hoechst, blue), neurons (NeuN, orange) and AAV transduced cells (GFP, green). The Harmony software pseudo-labels positive signals with a coloured border as shown in the lower right-hand side of the thumbnails. (**C**) Positive signals for NeuN and GFP were further gated based on user input and machine learning to remove false positives. A combination of these gated outputs were used to identify NeuN+, GFP+ and NeuN+/GFP+ cells. (**D**) Data from each optical slice throughout the Z-stack were combined to locate neurons and transduced cells throughout the entire section. (**E–G**) The percentage of the Hoechst+ cells found in each section that were NeuN+, GFP+ or both NeuN+/GFP+ for each mouse strain. **(H)** The automated analysis of the data collected from all brain sections was reanalyzed using less stringent gating criteria for GFP+. Allowing more cells to be registered as GFP+ increased the transduction efficiency in the C57BL/6J mice. However, a small percentage of the Hoechst+ cells in the CBA/Ca sections were also identified as being GFP+, indicating that this increased signal corresponded to background. Each data point is an individual brain section with each of the 3 C57BL/6J and 3 CBA/Ca mice delineated by distinctive symbols: circle, square or triangle. Scale bar = 1 mm for whole brain imaging, 25 μm for thumbnails.

To quantify AAV transduction within the brain, high-content automated imaging was used to capture signals across whole brain sections (Fig. 2A). A series of images were taken using a 40× lens and stitched together to capture all cells in an optical slice. A 10% overlap was used across all images to avoid loss of signal at the image periphery (Fig. 2A). Images were collected through the *Z*-axis at intervals of 1.2 μm with 19 images to encompass the whole thickness of the section (Fig. 2A). The 405 nm laser signal was captured to identify Hoechst staining, and Harmony’s inbuilt analysis was used to identify and segment cell nuclei (Fig. 2B). The signals from the 488 and 561 nm lasers were used to identify NeuN and GFP staining, respectively. The ‘find cells’ algorithm building block was then used to identify neurons (NeuN+) and AAV transduced cells (GFP+) (Fig. 2B).

To further segment true neurons and transduced cells from false positives the NeuN+ and GFP+ signals were further gated. The ‘Advanced STAR’ morphology analysis algorithm was applied for machine learning to identify true neurons or transduced cells, based on researcher input, and signals not meeting these new criteria were excluded (Fig. 2C). The outputs of true NeuN+ and GFP+ signals were combined with the Hoechst signal, before gating with the STAR morphology algorithm, to identify transduced neurons (NeuN+/GFP+) (Fig. 2C).

The outputs from individual optical slices were then combined with those captured through the *Z*-axis of a section (Fig. 2D). The final analysis of the Z-stack projection, accounted for cell-duplication between the optical slices, and produced a readout of all the cells within a brain section. The proportion of neurons, transduced cells and transduced neurons was then estimated for the whole brain by performing this quantification on every 12th section. There was no difference in the percentage of cells identified as neurons between the C57BL/6J and CBA/Ca mice, with approximately 40% of cells across the whole brain identified as neurons in both inbred strains (Fig. 2E). A GFP signal could be detected in approximately 14% of cells in the C57BL/6J brains. The estimated transduction rate between sections from the same mouse brain was highly comparable (Fig. 2F). These data indicated that one of the C57BL/6J mice (red circles) had a lower transduction efficiency of approximately 10% of cells. Between 10 and 15% of neurons had been transduced (Fig. 2G), with the same C57BL/6J mouse showing the lowest transduction efficiency (red circles).

In order to ensure that the exclusion criteria set by our automated analyses were not too stringent, and that the proportion of transduced cells had not been underestimated, the above analysis was repeated with less stringent gating criteria for GFP. Allowing more cells to be registered as GFP+ increased the transduction efficiency in the C57BL/6J mice (Fig. 2H); however, a small percentage of the Hoechst+ cells in the CBA/Ca sections were also identified as being GFP+. Since the ssAAV-PHP.B-CAG-NLS-GFP virus does not cross the BBB in CBA/Ca mice, these data could be used as an indication of increased background levels. Therefore, we consider that the original exclusion criteria were suitable for estimation of the ground-truth transduction efficiency.

### Segmentation of whole brain data by structural region

**Figure 3:**
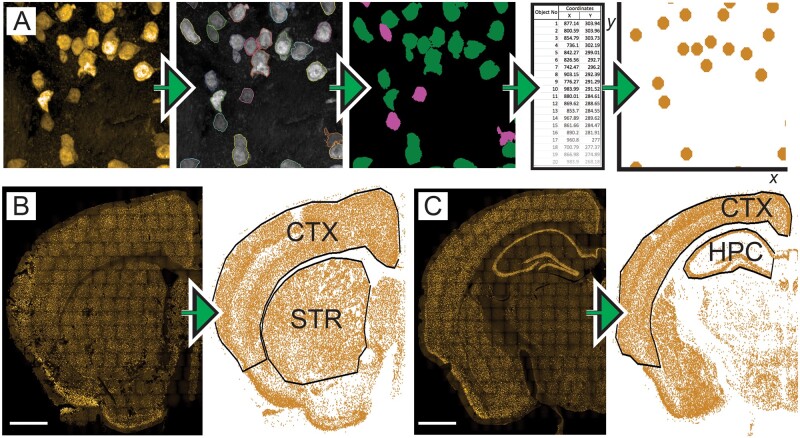
**Segmentation of whole brain data by structural region**. (**A**) Analysis of each image using the automated analysis tree allowed the identification of cells that were either NeuN+, GFP+ or NeuN+/GFP+. Each cell nucleus contained regional coordinate information relative to its position in the well. This information was exported as .csv files and graphed to recreate images on an axis. (**B–C**) This process was expanded to the whole brain composites, which allowed whole brain sections to be recreated as graphs. These graphs were manually segmented as regions of interest (black outlines) to allow transduction efficiencies in the cortex (CTX), striatum (STR) and hippocampus (HPC) to be estimated. Scale bar = 1 mm.

We next set out to develop an approach by which whole brain data could be segmented to determine the transduction efficiency of ssAAV-PHP.B-CAG-NLS-GFP by brain region. The automated analysis tree had allowed the identification of cells that were either NeuN+, GFP+ or NeuN+/GFP+ in each image. The Hoechst signal for each cell nucleus contained regional coordinate information relative to its position in the well. This information was exported as .csv files and the *x* and *y* coordinates were then used to recreate the brain section in a graphical format (Fig. 3A). The graphed output of the NeuN+ data was used to draw the ROIs, as it was easier to define brain regions without the non-neuronal cell bodies of the white matter tracts being present. The cortex, striatum and hippocampus were delineated by drawing around these ROIs using the gatepoints tool within R (Fig. 3B and C). Data within these areas were then exported to allow the proportions of neurons and transduction efficiencies to be determined.

### Estimation of AAVPHP.B transduction efficiencies in brain ROIs

**Figure 4:**
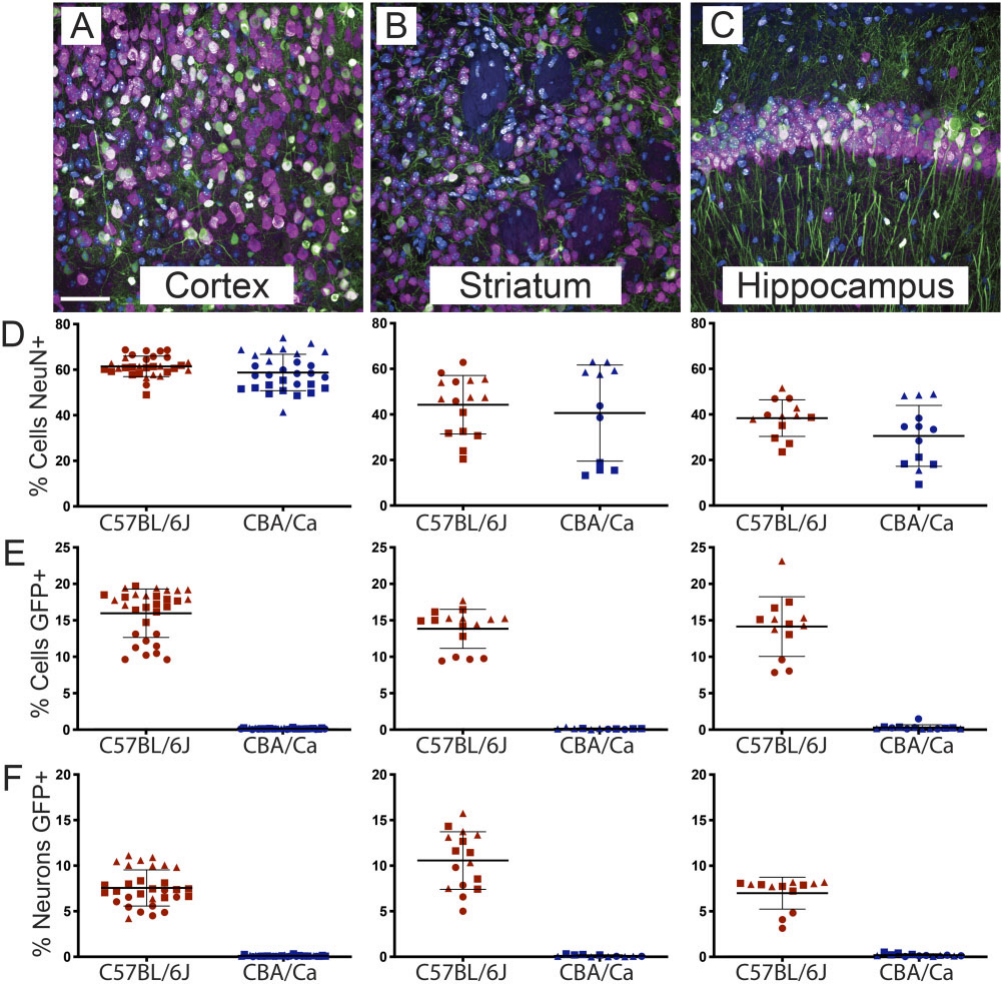
**Estimation of AAVPHP.B transduction efficiencies in brain regions of interest**. Regional information was extracted from images of the cortex, striatum and hippocampus using the graph segmentation approach described in Fig. 3. (**A–C**) Representative images of these regions stained with Hoechst and immunolabelled for NeuN and GFP. (**D**) The percentage of neurons in each brain region as indicated by the proportion of Hoechst+ cells that were NeuN+. (**E**) The percentage of transduced cells in each brain region as indicated by the proportion of Hoechst+ cells that were GFP+. (**F**) The percentage of transduced neurons in each brain region as indicated by the proportion of NeuN+ cells that were GFP+. Each data point represents a region of interest within a brain section, and each symbol (circle, square, triangle) represents one of the 3 C57BL/6J or 3 CBA/Ca mice. Mean ± SEM. Scale bar = 50 μm

We applied the brain region segmentation tool to compare the transduction efficiency of ssAAV-PHP.B-CAG-NLS-GFP in the cortex, striatum and hippocampus (Fig. 4). Consistent with the whole brain data, there was no difference in the percentage of neurons (NeuN+) between C57BL/6J and CBA/Ca mice in any brain region (Fig. 4B). In the cortex, NeuN+ neurons constituted around 60% of the Hoechst+ cells, whilst in the striatum and hippocampus this was closer to 40%. The striatal and hippocampal data were more variable between mice than the cortical data, but the percentage of NeuN+ cells was very consistent across sections from the same mouse brain for all ROIs (Fig. 4B).

Transduction rates of cells in the C57BL/6J mice were around 15% in all three brain regions (Fig. 4E), and in all cases a GFP signal was absent from the CBA/Ca mice. Consistent with the whole brain data, the transduction efficiency in one mouse (red circles), was lower than in the other two (Fig. 4E). The neuronal transduction efficiency in the cortex, striatum and hippocampus ranged between 5 and 15% (Fig. 4F).

### Biodistribution of AAV-PHP.eB virus 2 h after injection in brain and liver

**Figure 5:**
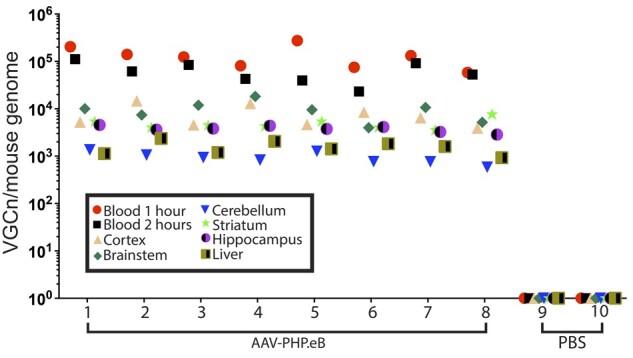
**Biodistribution of AAV-PHP.eB virus in brain and liver 2 h after intravenous injection**. C57BL/6J mice were intravenously injected with either ssAAV-PHP.eB-CAG-NLS-GFP (4 males and 4 females) or PBS (one male and one female). The number of viral genomes (VGCn) was determined in 10 ng genomic DNA extracted from blood taken at 1 and 2 h post-injection and from brain regions and liver at 2 h post-injection. PBS, phosphate buffer saline.

We next set out to investigate the extent to which the VGCn measured in blood might correlate with that present in brain and liver 2 h after dosing. C57BL/6J mice were injected with 1 × 10^11^ viral genomes of ssAAV-PHP.eB-CAG-NLS-GFP (*n* = 8) or with PBS (*n* = 2). The AAV-PHP.eB capsid confers a greater transduction efficiency of brain cells after intravenous injection than AAV-PHP.B.^4^ Blood was collected at 1 h post-injection. Mice were sacrificed after 2 h, blood was collected, and brain regions and liver were harvested after perfusion with PBS. The free ITR qPCR assay was used to estimate the VGCn/mouse genome for blood, liver and brain regions (Fig. 5). The differences in VGCn between blood, liver and specific brain regions was comparable between mice. The virus had entered the brain very rapidly, with the level in most brain regions being higher than that in liver after 2 h (Fig. 5). However, the between mouse variability of VGCn in blood at 1 or 2 h did not predict that in specific brain regions at 2 h post dosing (*P* > 0.05 for all VGCn correlations between blood and brain regions).

## Discussion

We have used the Opera Phenix HCS platform coupled with Harmony analytical software to develop an automated analysis pipeline to quantify AAV viral transduction throughout the mouse brain. This was applied to the AAV-PHP viruses that target the central and peripheral nervous systems noninvasively via intravenous delivery.^3–5^ Sections were stained with Hoechst, and antibodies to NeuN and GFP and the percentage of neurons, transduced cells and transduced neurons within a coronal section were calculated. We used R-studio and ‘gatepoints’ to segment the data captured from coronal brain sections into cortex, striatum and hippocampus. We found, that for a given mouse, the percentage of neurons and transduction efficiencies were highly comparable throughout the brain, and specific brain regions.

The Hoechst signal was used to identify all cell nuclei, and in this study, sections were immunostained with NeuN and GFP to identify neurons and transduced cells, respectively. The experiment was designed to sample all cells within a 25 μm section at 600 μm intervals, and each data point on the graphs represented a single coronal section. The R-scripts that we used to segment specific brain regions could equally be applied to sub-structures. Therefore, this approach provided an estimate of the parameters of interest throughout the whole brain, or an entire brain region. The intervals at which the coronal sections were sampled could be modified dependent on the brain structure, parameter under investigation or both. The AAV-PHP.B virus was intravenously administered to C57BL/6J mice that are permissive, and CBA/Ca mice that are non-permissive, for viral translocation across the BBB, and the CBA/Ca sections were used as background to ensure that the analysis-tree settings had estimated the ground-truth of transduction efficiency.

Estimation of the mean number of neurons (NeuN+/Hoechst) in sections from the whole brain was approximately 40% for both C57BL/6J and CBA/Ca mice, which when broken down by brain region was found to be approximately 60% for the cortex, consistent with previous estimates,^26^ and 40% for the striatum and hippocampus. In all cases, the NeuN+ percentages for each section from a given mouse tended to cluster. The cortical data were the most consistent between mice, whereas those for the striatum varied considerably. In the case of the CBA/Ca striatum, for example, ranging from less than 20% for one mouse to approximately 60% for another. This discrepancy is likely to have arisen due to tissue mounting variation. The striatum, as a structure, is harder to lie flat in the dish, under low melting point agarose, than the cortex, and whilst undulation does not impact on the capture of the very strong Hoechst signal, the detection of signals from the antibodies was not as robust. In general, the staining intensity achieved with a given antibody should be taken into consideration when designing a study.

The whole brain AAV-PHP.B transduction efficiency was very comparable between two C57BL/6J mice (∼15%) and lower in the third (∼10%) (Fig. 2F). For a given mouse, the transduction efficiency was very consistent in all sections throughout the whole brain, supported by the data for each of the brain regions. The transduction efficiencies were at the lower end of those previously reported for the administration of 1 × 10^11^ VGs of AAV-PHP.B, which ranged between 10% and 60% for both cortex and striatum,^4–6^ which could be accounted for by variation in the preparation of the virus. This automated approach has confirmed that delivery to the brain via the vasculature results in an even and widespread viral transduction, which is in stark contrast to that achieved via intracerebral injection.^27^

The free-ITR quantitative Taqman qPCR assay was used to determine the extent to which the VGCn as measured in blood, might predict viral delivery to the brain. We measured VGCn in blood taken at 1 and 2 h post-injection with AAV-PHP.eB, and in liver and brain regions after 2 h. The virus had entered the brain very rapidly, with the level in brain being comparable to that measured previously in a similar experiment.^8^ VGCn levels in most brain regions were greater than that in liver, in keeping with the previous report.^8^ The comparative levels in VGCn between the blood, liver and various brain regions was very similar between mice. Within the brain, the highest levels were consistently seen in cortex and brain stem, with the lowest levels in cerebellum. Whether this translates to a higher level of neuronal transduction in brain stem as compared to cerebellum would require further investigation. The VGCn variability between mice for a given brain region was comparatively low; and there was no correlation with the variability seen in blood taken at either 1 or 2 h after dosing with that in specific brain regions. However, measuring the VGCn in blood shortly after dosing should still be performed to provide assurance of viral delivery on a gross level.

This automated analysis-tree provides an invaluable tool with which to rapidly assess the tropism of newly engineered AAV capsids. The repertoire of antibodies can be selected to investigate the transduction efficiencies of designated cells in the brain or different tissues, and the frequency at which tissue sections are sampled can be modified, to identify any variability in transduction efficiencies throughout a segmented ROI. The application of this analysis, to confirm the tropism and transduction efficiency of viral preparations in preclinical gene therapy experiments, through the addition of a small tag (e.g. HA or FLAG), will provide important quality control data to support efficacy studies. In a wider context, the methodology developed here will help to decrease bias and automate the quantification of any parameter of interest that can be captured as a fluorescent signal.

## Abbreviations

AAV =: adeno-associated virus
BBB =: blood brain barrier
CREATE =: Cre-recombination-based AAV targeted evolution
CT =: common threshold
FELASA =: Federation of European Laboratory Animal Science Associations
GFP =: green fluorescent protein
HCS =: high-content screening
IT =: individual threshold
ITR =: inverted terminal repeat
NLS =: nuclear localization signal
PBS =: phosphate buffered saline
PFA =: paraformaldehyde
qPCR =: real-time quantitative PCR
ROI =: region of interest
SC =: splitting coefficient
VGCn =: viral genome copy number
